# Required efficacy for novel therapies in BCG‐unresponsive non‐muscle invasive bladder cancer: Do current recommendations really reflect clinically meaningful outcomes?

**DOI:** 10.1002/cam4.2980

**Published:** 2020-03-12

**Authors:** Marian S. Wettstein, David Naimark, Thomas Hermanns, Jaime O. Herrera‐Caceres, Ardalan Ahmad, Michael A.S. Jewett, Girish S. Kulkarni

**Affiliations:** ^1^ Division of Urology Department of Surgery Princess Margaret Cancer Centre University Health Network University of Toronto Toronto Ontario Canada; ^2^ Department of Urology University Hospital of Zurich University of Zurich Zurich Switzerland; ^3^ Division of Nephrology Department of Medicine Sunnybrook Health Sciences Centre Toronto Ontario Canada

**Keywords:** BCG vaccine, clinical trial, phase ii, computer simulation, cystectomy, decision support techniques, organ sparing treatments, urinary bladder neoplasms

## Abstract

**Background:**

Single‐arm trials are currently an accepted study design to investigate the efficacy of novel therapies (NT) in non‐muscle invasive bladder cancer (NMIBC) unresponsive to intravesical Bacillus Calmette‐Guérin (BCG) immunotherapy as randomized controlled trials are either unfeasible (comparator: early radical cystectomy; ERC), or unethical (comparator: placebo). To guide the design of such single‐arm trials, expert groups published recommendations for clinically meaningful outcomes. The aim of this study was to quantitatively verify the appropriateness of these recommendations.

**Methods:**

We used a discrete event simulation framework in combination with a supercomputer to find the required efficacy at which a NT can compete with ERC when it comes to quality‐adjusted life expectancy (QALE). In total, 24 different efficacy thresholds (including the recommendations) were investigated.

**Results:**

After ascertaining face validity with content experts, repeated verification, external validation, and calibration we considered our model valid. Both recommendations rarely showed an incremental benefit of the NT over ERC. In the most optimistic scenario, an increase in the IBCG recommendation by 10% and an increase in the FDA/AUA recommendation by 5% would yield results at which a NT could compete with ERC from a QALE perspective.

**Conclusions:**

This simulation study demonstrated that the current recommendations regarding clinically meaningful outcomes for single‐arm trials evaluating the efficacy of NT in BCG‐unresponsive NMIBC may be too low. Based on our quantitative approach, we propose increasing these thresholds to at least 45%‐55% at 6 months and 35% at 18‐24 months (complete response rates/recurrence‐free survival) to promote the development of clinically truly meaningful NT.

## Graphical abstract

Expert groups published recommendations regarding clinically meaningful outcomes for single‐arm trials evaluating the efficacy of novel therapies in BCG‐unresponsive non‐muscle invasive bladder cancer. In this study, we used a discrete event simulation framework in combination with a supercomputer to demonstrate that these recommendations do probably not reflect clinically meaningful outcomes and should be increased to at least 45%‐55% at 6 months and 35% at 18‐24 months (complete response rates/recurrence‐free survival).

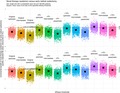

## 1 INTRODUCTION

Patients with non‐muscle invasive bladder cancer (NMIBC) unresponsive to intravesical Bacillus Calmette‐Guérin (BCG) immunotherapy pose a clinical dilemma^1^: On the one hand, surgical tumor extirpation before progression to muscle‐invasive disease (ie, early radical cystectomy; ERC) is the sole standard therapy.^1^, ^2^ However, this procedure is not only associated with considerable perioperative mortality and morbidity^3^ but also leads in most patients to a substantial decrease in quality of life.^4^ On the other hand, several bladder‐sparing novel therapies ranging from systemic immune checkpoint blockade to photodynamic therapy have been proposed during the last years.^1^ Although these modalities can potentially delay or even avoid ERC and hence preserve quality of life, they all come at the cost of inferior cancer control.

Considering the infeasibility of a randomized controlled trial comparing a specific novel therapy to ERC, the unavailability of an effective comparator arm besides ERC, and the fact that it is unethical to use placebo controls in this setting, the United States Food and Drug Administration (FDA) currently allows single‐arm phase II trials for the assessment and registration of novel therapies in BCG‐unresponsive NMIBC.^5^ Two recommendations regarding clinically meaningful outcomes of such single‐arm trials have been published so far: The International Bladder Cancer Group (IBCG) considers complete response rates (carcinoma in situ; CIS) and recurrence‐free rates (papillary tumors) of 50% at 6 months, 30% at 12 months, and 25% at 18 months as clinically meaningful^6^ while a public workshop of the FDA and the American Urological Association (AUA) proposed 40%‐50% at 6 months and 30% at 18‐24 months (regardless if CIS or papillary tumor).^7^ Although both recommendations are products of intensive discussions among renowned experts, they are not purely based on quantitative evidence. Therefore, the aim of this study was to quantitatively verify the two recommendations using a decision‐analytic approach. Specifically, we used a simulation framework in combination with the infrastructure of a supercomputer to find the required efficacy at which a novel therapy can compete with ERC when it comes to quality‐adjusted life expectancy.

## 2 METHODS

We built a discrete event simulation model in the statistical programing language R (R Core Team, Vienna, Austria). The simulation‐specific framework was implemented by the *simmer* package, a package specifically developed for discrete event simulations in R.^8^ Computations were performed on the Niagara supercomputer using a 640‐core setup (SciNet HPC Consortium, Toronto ON, Canada).^9^, ^10^ We followed the methodology described by Caro* et al* ^11^ during model development, validation, calibration, and analysis. The reporting of this study is in accordance with the guidelines published by the Society of Medical Decision Making.^12^ The Appendix S1 provides an in‐depth description of the model.

### 2.1 Simulation setting and strategies

We simulated different management strategies (including ERC and novel therapies) in a cohort of BCG‐unresponsive NMIBC patients from the date of diagnosis of BCG‐unresponsive disease to the date of death. Age, gender, and tumor type (ie, CIS, Ta, Ta + CIS, T1, T1 + CIS) were sampled based on recent trials in the BCG‐unresponsive NMIBC setting.^13^, ^14^ The strategy “ERC” consisted of immediate performance of ERC and potential adjuvant chemotherapy (based on local tumor extension, nodal status, and patient preference). As the term “novel therapy” stands for various treatment modalities that differ with regard to route of administration, duration and adverse effects, we decided to represent “novel therapy” by three distinct strategies (ie, systemic, low‐intensity intravesical, high‐intensity intravesical) that share several characteristics as described in Table 1.

**TABLE 1:** Characterization of the three types of novel therapies that were all simulated as distinct strategies

Type of novel therapy	Systemic	Low‐intensity intravesical	High‐intensity intravesical
Description	Immune checkpoint blockade or targeted therapy	Local therapy with a low toxicity compound, usually several applications	Local therapy with a high toxicity compound, usually a single application
Typical example	Pembrolizumab (every 3 weeks for 2 years)	rAd–IFNa/Syn3 (4 applications distributed over 1 year)	Photodynamic therapy (single application)
Assumed duration^a^	2 years	1 year	3 months
Burden of systemic adverse effects	Moderate/high	Low	Low
Burden of urogenital adverse effects	Low	Low/moderate	Moderate/high

^a^ This duration reflects the whole active treatment phase during which we assumed the disutility of the novel therapy to be present.

Regardless of the specific type of novel therapy, all of the strategies involving novel therapy start with the initial treatment application. Patients are then followed with cystoscopy and cytology (intervals of 3 months in year 1 and 2, 6 months in years 3 and 4, 12 months after year 4) until they die from a cause unrelated to bladder cancer or until they experience failure of the novel therapy. In the event of treatment failure, patients receive a standardized workup (transurethral resection/bladder biopsy and cross‐sectional imaging) before they proceed to radical cystectomy. Depending on the staging results, patients might be offered neoadjuvant chemotherapy (in the presence of muscle‐invasive disease) or the surgeon might cancel radical cystectomy (in the presence of metastatic progression). From the time point of radical cystectomy, patients of strategies involving novel therapies share a common trajectory with the patients of the strategy “ERC” and can therefore also receive adjuvant chemotherapy (depending on local tumor extension, nodal status, patient preference, and under the provision that no neoadjuvant chemotherapy was given). In the post‐cystectomy phase, patients can die due to causes unrelated to bladder‐cancer or they might experience disease recurrence. In the event of disease recurrence, they receive maximal palliative treatment until they die from bladder cancer.

### 2.2 Simulation logic

A discrete event simulation is structured around events. Events can represent clinical events such as cystoscopy/cytology visit or death but also biological events such as progression to muscle‐invasive disease or development of distant metastases. Based on specified time to event distributions, each patient gets a distinct set of event times. During the actual simulation, a patient always experiences the event that is next in time (eg, start of adjuvant chemotherapy is experienced before recurrence if time_recurrence_ = 5 years and time_start adjuvant chemotherapy_ = 10 weeks). It should also be noted that experiencing a certain event can lead to the modification of other event times (eg, multiplication of time_recurrence_ by factor 1.3 after completion of adjuvant chemotherapy). After all patients have died, which terminates the simulation, the quality‐adjusted life expectancy (QALE) of each individual can be reconstructed by weighting different periods of the clinical course with health state utility values (0: death; 1: perfect health). As an example, a patient who had lived 5 years in perfect health and 5 additional years with bothersome symptoms (utility: 0.7) before she finally died would accumulate 5 × 1 + 5 × 0.7 = 8.5 quality‐adjusted life years. By averaging the accumulated QALE of each strategy, it is possible to compare two or more strategies such as ERC and different types of novel therapies.

**FIGURE 1:**
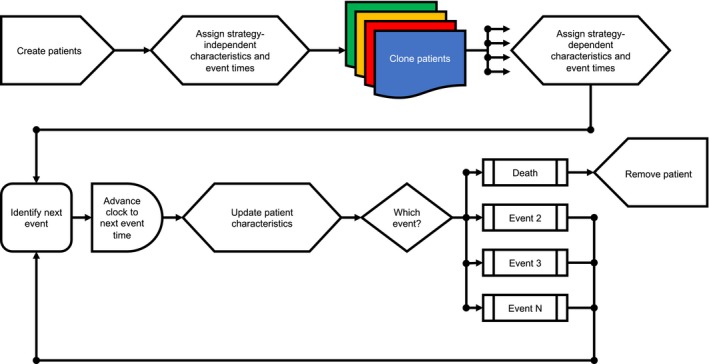
Simulation logic

Figure 1 visualizes the logic of our discrete event simulation model. In a first step a cohort of hypothetical patients is created. Next, specific characteristics (such as age, sex, and tumor type) as well as strategy‐independent event times are assigned to all individuals before the cohort is cloned four times (one clone per strategy; ie, ERC and three types of novel therapies). The cloning can not only be regarded as the modeling equivalent to randomization in a randomized controlled trial (as it creates strategy arms with identical baseline characteristics) but also as a means to reduce unwanted variance.^11^, ^12^ After cloning but before the start of the simulation, patients receive strategy‐dependent modifications of characteristics and event times. As soon as the simulation clock runs, the simulation logic identifies the first event among all individuals (eg, start of radical cystectomy in patient 12 in 3 days). The simulating clock is then advanced to the identified event time (eg, 3 days), the identified patient (eg, patient 12) gets an update of certain characteristics (eg, age + 3 days), and also experiences the identified event (eg, start of radical cystectomy), which might include the modification of characteristics or event times. After processing this event, the simulation logic identifies the next event and so on. Individuals that experience the event “death” leave the simulation. A detailed description of the full simulation logic can be found in the Appendix S1.

### 2.3 Model input

Our simulation required different types of input parameters such as event times (eg, time to death), probabilities (eg, mortality during radical cystectomy), and health state utility values (eg, health state utility of receiving perioperative chemotherapy) that were mostly obtained through systematic literature search. Event times were sampled from either Weibull distributions, exponential distributions, or uniform distributions. Life tables provided by the National Center for Health Statistics of the United States served as source data to sample the background mortality of each patient based on age and sex.^15^ Whenever possible, we used published multivariable regression models^16^, ^17^, ^18^, ^19^ to inform model inputs based on patient characteristics, which allowed us to, for example, sample time to tumor recurrence after radical cystectomy based on sex, age, tumor characteristics, and perioperative chemotherapy. Two input parameters (median time to muscle invasion after failure of novel therapy and median time to metastatic progression after muscle invasion) could not be derived through literature review and plausible ranges had to be estimated through expert opinion (bounded by extrapolations from related but not readily comparable literature). To account for the uncertainty of these two parameters as well as for the uncertainty associated with health state utility values, we performed probabilistic analyses and therefore replicated the simulation 1000 times, each time with a different set of plausible input parameters. A detailed list of all input parameters (including the methodology used for their derivation) can be found in the Appendix S1.

### 2.4 Validation and calibration

The validity of our model was iteratively ascertained during the whole research process. Face validity (level of model complexity, plausibility of clinical patient trajectories, consistency of outcomes) was verified by involving content experts at all stages of development. In addition, the whole model was formally debugged several times to detect potential coding errors and/or inconsistencies. Furthermore, we externally validated the simulation output by comparing the predicted outcomes with results published in literature (validation targets). Discrepancies between validation targets and model output were addressed by calibrating certain input parameters assumed to be influential (calibration input). Specifically, this involved repeating the simulation thousands of times while varying the calibration input. The calibrated values were then derived by retaining the calibration input sets that yielded minimal discrepancy between model output and validation targets. In accordance with prior work of Vanni* et al*,^20^ we included the best fitting values from the calibration process in the probabilistic analysis to account for the uncertainty associated with them. A detailed description of the external validation as well as of the calibration process can be found in the Appendix S1.

### 2.5 Analysis

To quantitatively verify if the recommendations issued by the IBCG and the FDA/AUA reflect clinically meaningful outcomes from a QALE perspective, we fixed the parameters of the time to event distribution reflecting “time to failure novel therapy” to values that mirror the efficacy statements “50% at 6 months, 30% at 12 months, and 25% at 18 months” (IBCG) and “40%‐50% at 6 months and 30% at 18‐24 months” (FDA/AUA). We therefore used an optimization algorithm (simulated annealing^21^) to find piece‐wise exponential distributions that match the above‐mentioned value pairs as close as possible. Since these recommendations do not specify the efficacy beyond two years, it was necessary to assume three scenarios that we defined as follows: optimistic behavior (ie, treatment failure in additional 5% of the patients until year 5, in additional 1% between 5 years and plateau at 7.5 years), intermediate behavior (ie, additional 5% until year 5, additional 5% between 5 years and plateau), and pessimistic behavior (ie, additional 10% until year 5, additional 5% between 5 years and plateau). Furthermore, we explored higher efficacy thresholds by increasing the two recommendations by +5%, +10%, and + 15% (each with optimistic, intermediate, and pessimistic behavior after the initial two years of treatment). The resulting 24 failure curves are visualized in the Appendix S1.

To account for all nuances of our research question, the final analysis had to involve three simulation levels (ie, lower, middle, upper): At the lower level, each of the four strategies was simulated among a cohort of 100,000 patients. We aggregated the discounted (3%) QALE of the resulting 400,000 individuals (4 strategies × 100,000 patients) into strategy‐specific means. Next the incremental benefit of each type of novel therapy was defined as the difference between QALE_Novel therapy_ and QALE_ERC_. We empirically chose a cohort size of 100,000 patients as this number yielded highly stable results with a percentage deviation of less than 1% from the mean value. At the middle level, we replicated, as described earlier, each lower‐level run 1000 times with probabilistic input parameters and aggregated the results into means to reflect the uncertainty associated with some input parameters (expert opinions, health state utility values, and parameters derived through calibration). Last, we simulated at the upper level the 24 above‐mentioned efficacy thresholds. Therefore, reliably analyzing 24 efficacy thresholds required simulating the clinical course of 9.6 billion individuals (4 strategies × 100,000 patients × 1000 probabilistic samples × 24 efficacy thresholds).

## 3 RESULTS

### 3.1 Validity of model

**FIGURE 2:**
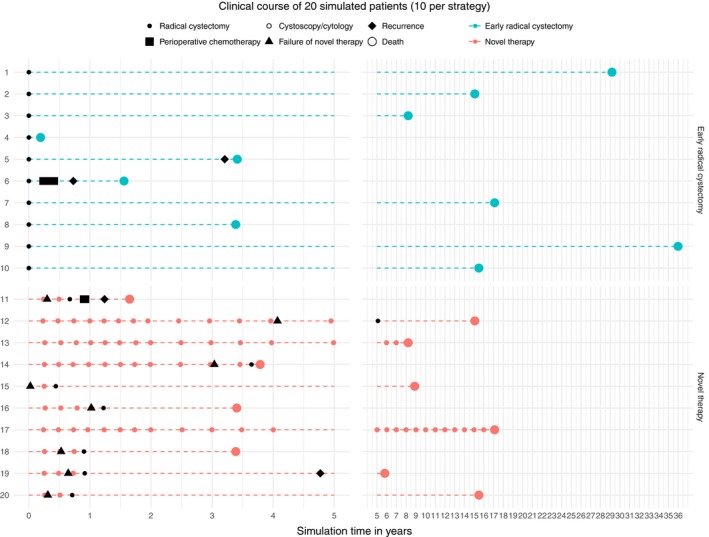
Clinical course of 20 simulated patients (10 per strategy)

After ascertaining face validity with content experts, repeated verification, external validation, and calibration we considered our model valid. Figure 2 visualizes the clinical course of 20 random patients that were generated by our simulation at an efficacy threshold reflecting an optimistic interpretation of the IBCG recommendation. The upper 10 patients were managed by the strategy “ERC” while the lower 10 patients received the strategy “Novel therapy.” It can be seen that even this simplified visualization of our model has enough complexity to account for clinical events such as perioperative chemotherapy (patients 6 + 11) and death as a complication of radical cystectomy (patients 4 + 14). After calibration, external validation of the strategy “ERC” against an ERC cohort^22^ that was not used as input source demonstrated credible results (simulation output vs external source) regarding pT3/pT4 disease at ERC (15.4% vs 13%), positive nodal disease at ERC (13.2% vs 13%), and cancer‐specific survival (86.6% vs 85% at 5 years, 76.4% vs 76% at 10 years).

### 3.2 Required efficacy of novel therapies

**FIGURE 3:**
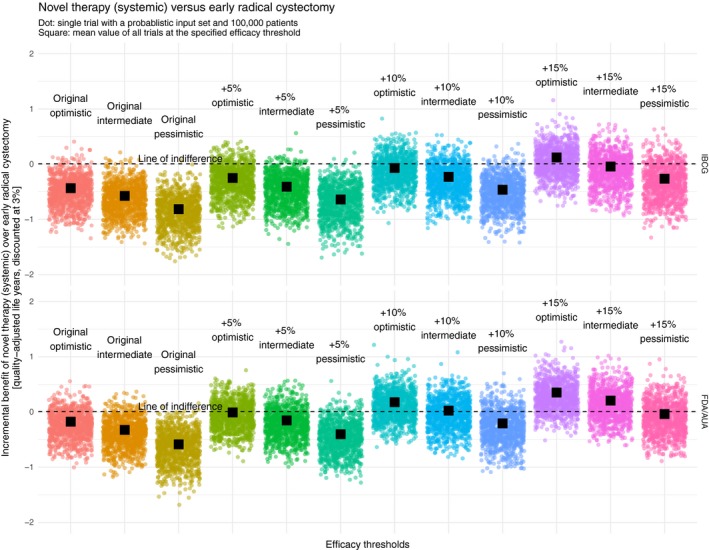
Incremental benefit of novel therapy (systemic) over early radical cystectomy [quality‐adjusted life years, discounted at 3%]. AUA, American Urological Association; FDA, United States Food and Drug Administration; IBCG, International Bladder Cancer Group

**FIGURE 4:**
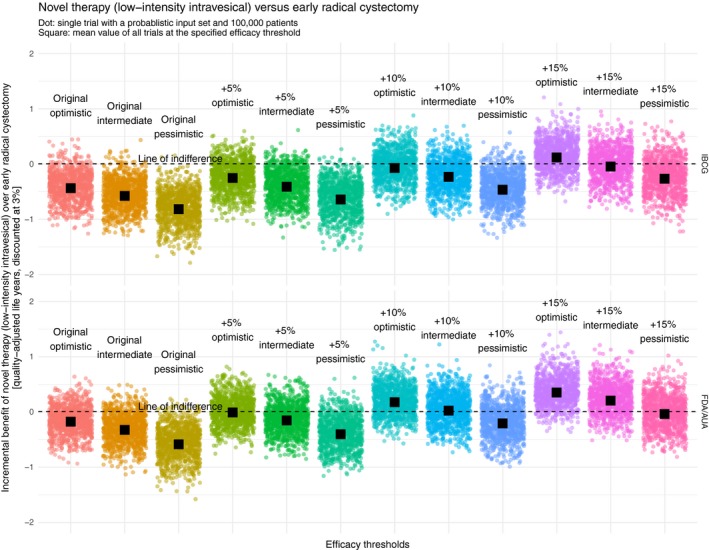
Incremental benefit of novel therapy (low‐intensity intravesical) over early radical cystectomy [quality‐adjusted life years, discounted at 3%]. AUA, American Urological Association; FDA, United States Food and Drug Administration; IBCG, International Bladder Cancer Group

**FIGURE 5:**
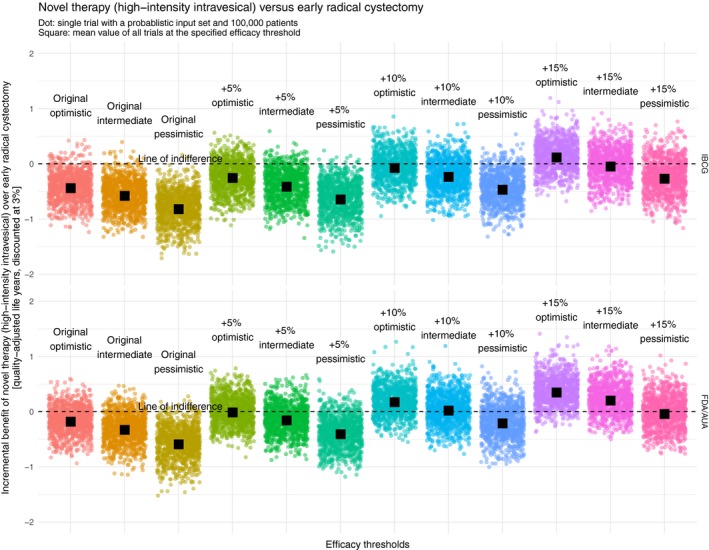
Incremental benefit of novel therapy (high‐intensity intravesical) over early radical cystectomy [quality‐adjusted life years, discounted at 3%]. AUA, American Urological Association; FDA, United States Food and Drug Administration; IBCG, International Bladder Cancer Group

We visualized the incremental benefit of the novel therapies “systemic,” “low‐intensity intravesical,” and “high‐intensity intravesical” over the reference strategy “ERC” at different efficacy thresholds in Figures 3, 4, 5 respectively. Both the original IBCG recommendation and the original FDA/AUA recommendation rarely showed an incremental benefit of the novel therapies over ERC. Especially when looking at an optimistic interpretation of the IBCG recommendation, it can be seen that only 2.4%, 5.6%, and 5.1% of all probabilistic samples favor the novel therapies “systemic, “low‐intensity intravesical,” and high‐intensity intravesical,” respectively. Although an optimistic interpretation of the FDA/AUA recommendation yields considerably better results of 16.0%, 27.0%, and 25.7% of simulations, the resulting means are still below the line of indifference.

To find a valid recommendation at which a novel therapy can compete with ERC from a QALE perspective, it is necessary to identify an efficacy threshold at which the probabilistic results evenly spread around a mean of zero with ideally 50% of all sets favoring the novel therapy. When looking at variations of the IBCG recommendation, the point of indifference is reached, regardless of the specific type of novel therapy, between +10% and 15% (optimistic), at 15% (intermediate), and much higher than 15% (pessimistic). Replicating this approach among variations of the FDA/AUA recommendation, indifference is achieved, again regardless of the specific type of novel therapy, at 5% (optimistic), 10% (intermediate), and 15% (pessimistic).

## 4 DISCUSSION

In this simulation study, we quantitatively verified if current recommendations regarding clinically meaningful outcomes in single‐arm trials evaluating novel therapies in BCG‐unresponsive NMIBC translate into meaningful outcomes from an integrative healthcare perspective. We therefore compared three different types of novel therapies against ERC as the standard of care. Our work not only showed that there is a low probability that the recommendation of the IBCG (50% at 6 months, 30% at 12 months, and 25% at 18 months) or the one of the FDA/AUA (40%‐50% at 6 months and 30% at 18‐24 months) would make any novel therapies a real competitor to ERC but also that these recommendations need to be increased by at least 10% (IBCG) and 5% (FDA/AUA). Thus, we feel that an appropriate recommendation should suggest an efficacy threshold of at the minimum 45%‐55% at 6 months and 35% at 18‐24 months (complete response rate/recurrence‐free survival).

Effective novel therapies for BCG‐unresponsive NMIBC are desperately needed. Clinician‐scientists and companies embarking on those single arm phase II registration trials therefore need guidance regarding appropriate designs and clinically meaningful outcomes. Since there is a low probability that the current recommendations translate into competitive outcomes, meaning that the novel therapy will yield on average the same amount or even more quality‐adjusted life years than ERC, there is also a low probability that the resulting innovation will be cost‐effective. Hence, to prevent the development of cost‐ineffective novel therapies, it is of high importance to increase these recommendations to levels where novel therapies yield at least a QALE comparable to ERC. It should be noted, however, that although our simulation recommends a clinically meaningful efficacy threshold for such single‐arm trials, it does not inform on sample sizes that would be required to yield acceptable statistical reliability of the efficacy estimate.

In January 2020, the FDA approved pembrolizumab, a monoclonal antibody targeting the programmed cell death protein 1 (PD‐1), for the treatment of patients with BCG‐unresponsive, high‐risk, NMIBC with CIS with or without papillary disease who are ineligible for or have elected not to undergo ERC.^23^ The decision was largely based on interim results (N = 96; cohort A) of the KEYNOTE‐057 study, a single‐arm phase II trial, that demonstrated a complete response rate of 41% (95% confidence interval: 31%‐51%) and a complete response duration over one year in 20% of all patients.^24^ Although the investigators used a slightly different outcome measure, it is evident that the current KEYNOTE‐057 results do not meet our proposed efficacy thresholds and that their clinical meaningfulness has to be doubted. The FDA’s 9:4 vote in favor of pembrolizumab^23^ despite unclear clinical meaningfulness was probably mostly driven by the unmet clinical need and the fact that pembrolizumab outperformed prior less successful NTs such as valrubicin by far.^25^ Furthermore, the approval has to be interpreted also in the light of the developments during the last two decades: As investigated by Tibau* et al*, the FDA approved 27 cancer drugs between 2006 and 2016 based on single‐arm trials although only a minority of them (7.4%) met the threshold for substantial clinical benefit as measured by the European Society for Medical Oncology Magnitude of Clinical Benefit Scale.^26^, ^27^

This study is novel as it leverages the power of a decision‐analytic approach by using the infrastructure of a supercomputer. This allowed us to not only investigate 24 different efficacy thresholds, but also to explore the uncertainty associated with each threshold by 1000 probabilistic input sets. At the same time, we were able to simulate 400,000 patients per probabilistic run which yielded highly stable results. Such a powerful approach would not have been feasible in the setting of a personal computer.

Nevertheless, our work has an important limitation: It is at the end of the day a simulation of hypothetical patients and might therefore be suspiciously regarded as a “black box.” We tried to mitigate this suspicion at several levels. First, we extensively ascertained the validity of our model which included, besides other steps, calibration against data from the literature. Second, we explored uncertainty using 1000 probabilistic input sets per threshold. Lastly, we tried to provide full transparency by making the model documentation readily available in the Appendix S1.

This simulation study demonstrated that the current recommendations regarding clinically meaningful outcomes for single‐arm phase II registration trials evaluating the efficacy of novel therapies in BCG‐unresponsive NMIBC are too low. There is a high probability that novel therapies with an efficacy as low as specified by the IBCG and FDA/AUA cannot compete with the ERC and therefore will never be clinically efficient from an integrated healthcare perspective. Based on our quantitative approach, we propose increasing these thresholds to at least 45%‐55% at 6 months and 35% at 18‐24 months (complete response rate/recurrence‐free survival) to promote the development of clinically truly meaningful novel therapies.

## CONFLICT OF INTEREST

The authors have no conflict of interest to declare.

## AUTHOR CONTRIBUTIONS

Marian S. Wettstein contributed to conceptualization, data curation, formal analysis, funding acquisition, investigation, methodology, project administration, software, visualization, writing – original draft, and writing – review and editing. David Naimark and Girish S. Kulkarni contributed to conceptualization, methodology, supervision, and writing – review and editing. Thomas Hermanns, Jaime O. Herrera‐Caceres, Ardalan Ahmad, and Michael AS Jewett contributed to conceptualization, writing – review and editing.

## Supporting information

Supplementary Material

## Data Availability

The source code is available on request from the authors.
