# Supplementary material for: Required efficacy for novel therapies in BCG‐unresponsive non‐muscle invasive bladder cancer: Do current recommendations really reflect clinically meaningful outcomes?
Source: Cancer Med. 2020 Mar 12;9(10):3287–96. doi: 10.1002/cam4.2980 (PMC7221312; doi:10.1002/cam4.2980)
Supplement: Supplementary file 1 — Supplementary Material [file CAM4-9-3287-s001.pdf]

Supplemental Appendix

The source code is available on request from the authors.

Influence diagrams 2
Assumptions 5
Simulation logic 6
Input parameters 10
  Patient characteristics at start of simulation 10
  Time to event parameters 10
  Probabilities 15
  Utilities 16
Validation and calibration 17
  External validation methodology 17
  Calibration methodology 17
Failure curves 20
Supercomputer configuration 21
References 23

Influence diagrams

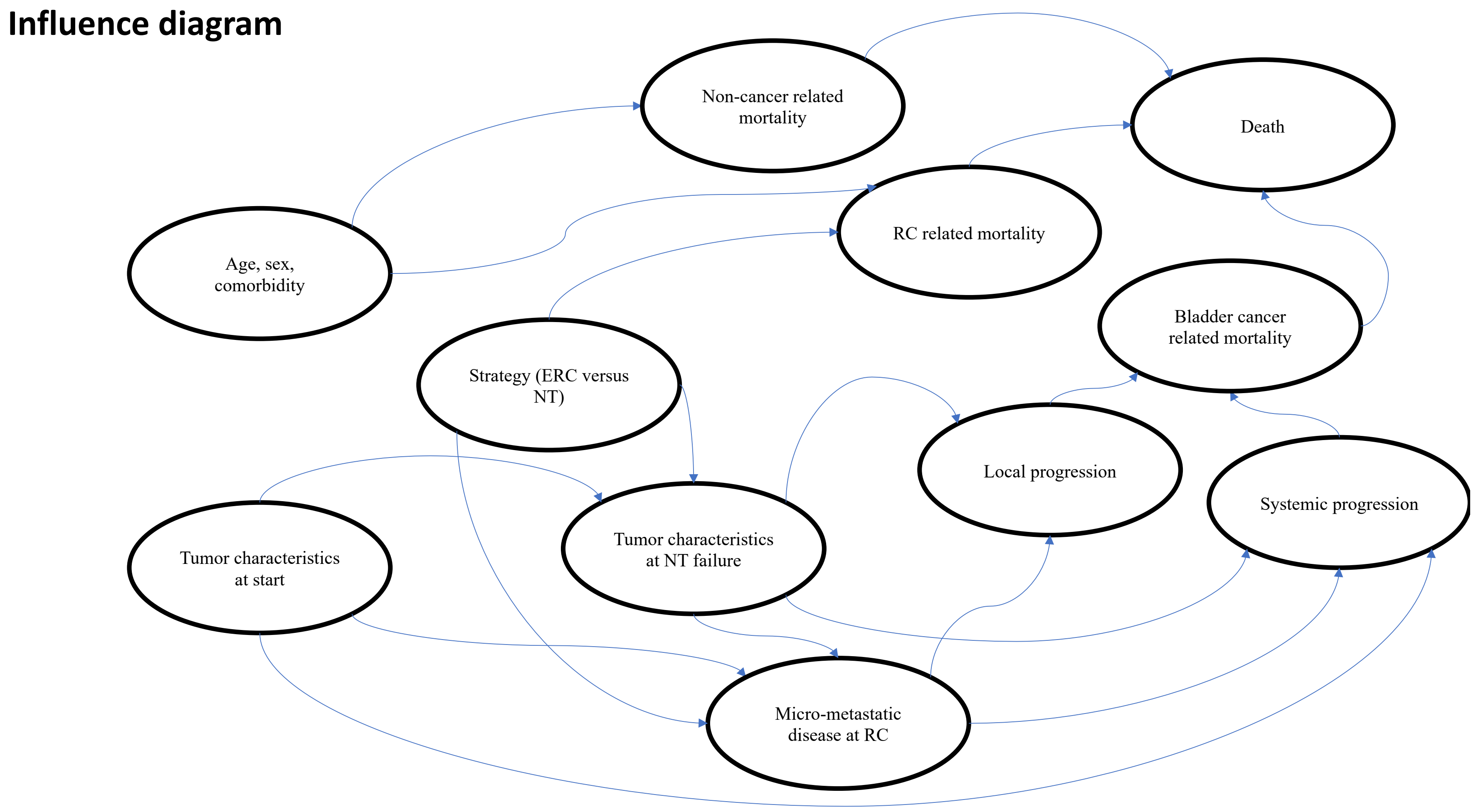

Supplemental Figure 1: Influence diagram visualizing the concepts incorporated into the discrete event simulation model and their assumed interaction. Arrows represent influences but do not imply causality. *ERC: early radical cystectomy; NT: novel therapy; RC: radical cystectomy*

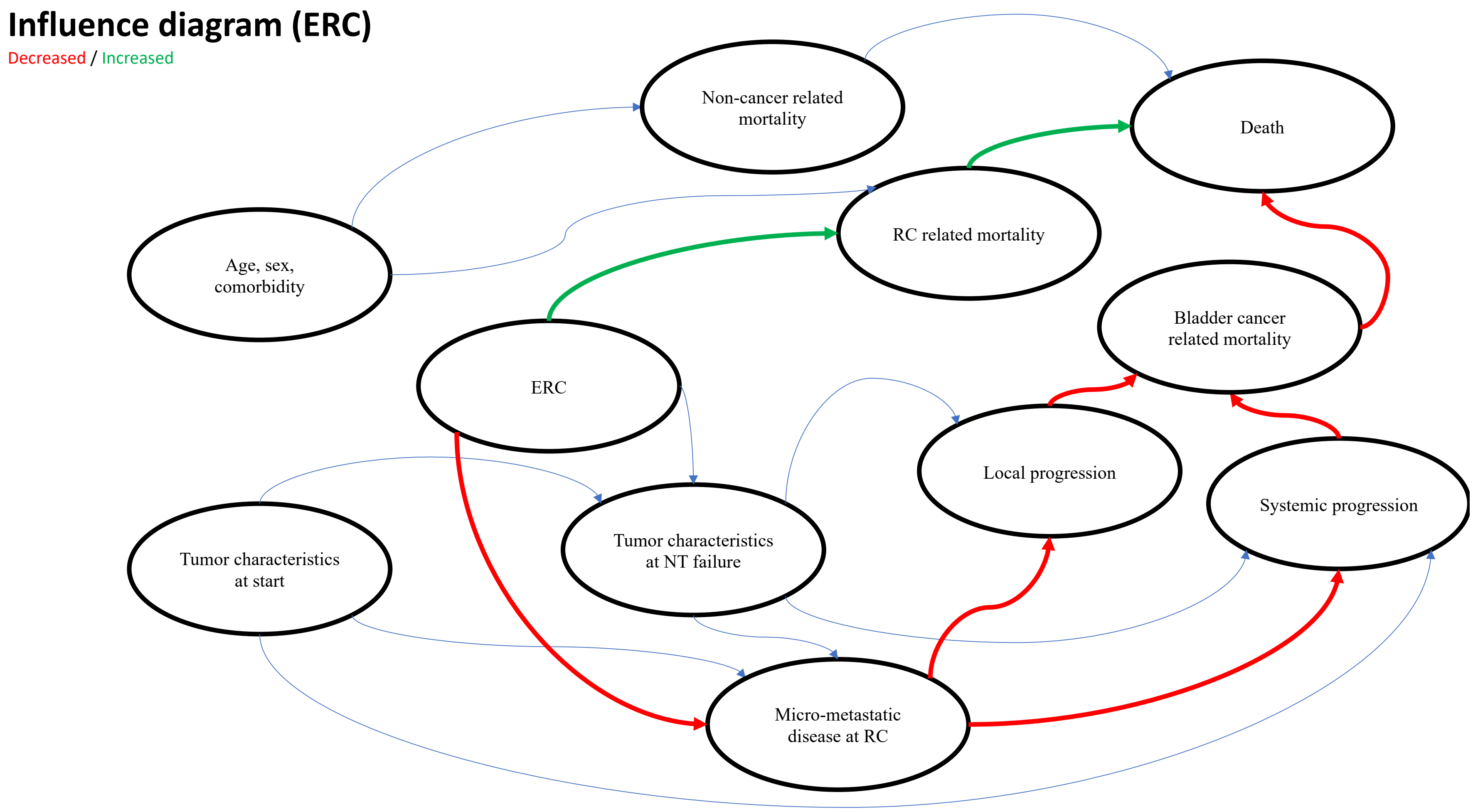

Supplemental Figure 2: Influence diagram visualizing the concepts incorporated into the discrete event simulation model and their assumed interaction (strategy: early radical cystectomy). Arrows represent influences but do not imply causality. *ERC: early radical cystectomy; NT: novel therapy; RC: radical cystectomy*

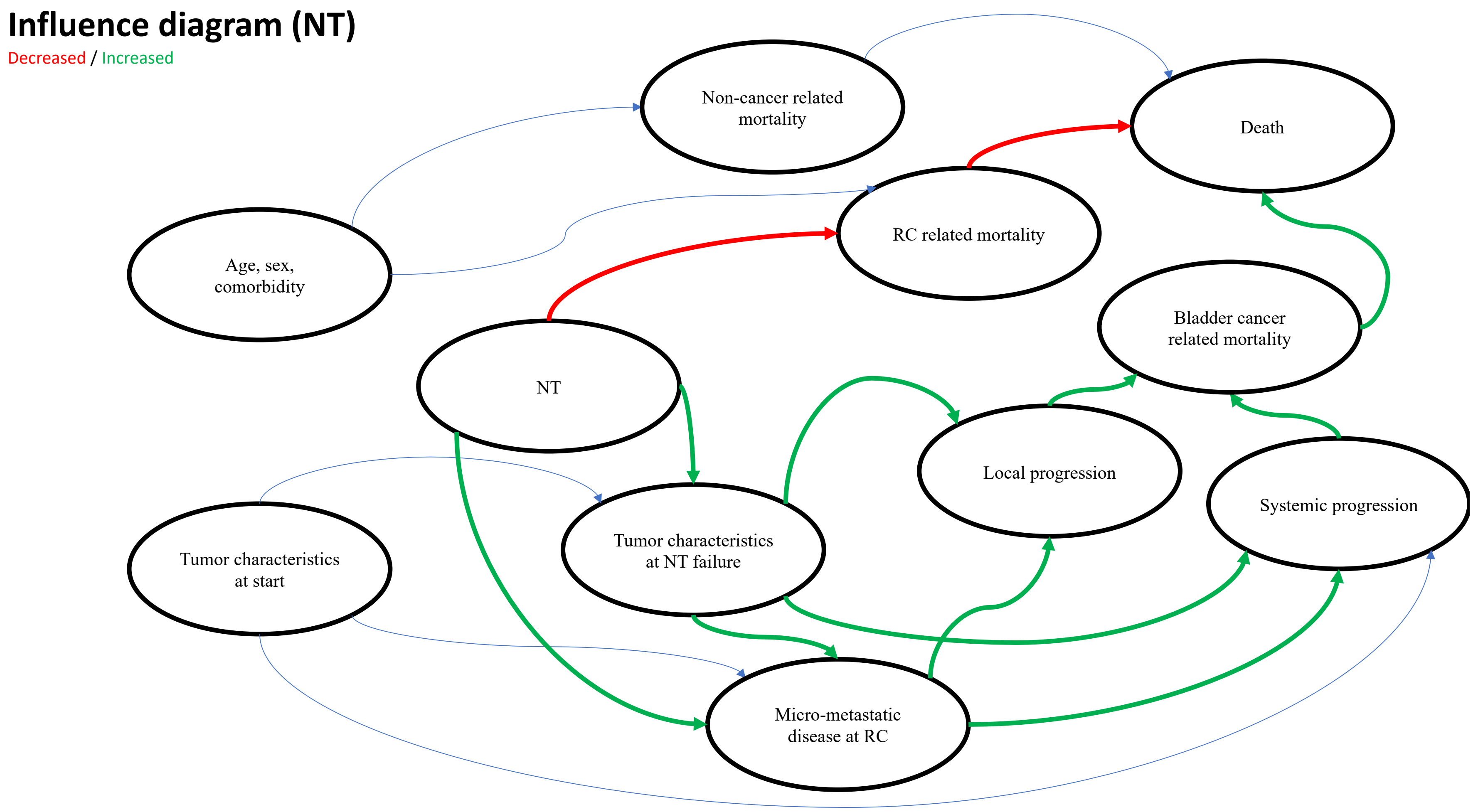

Supplemental Figure 3: Influence diagram visualizing the concepts incorporated into the discrete event simulation model and their assumed interaction (strategy: novel therapy). Arrows represent influences but do not imply causality. *ERC: early radical cystectomy; NT: novel therapy; RC: radical cystectomy*

Assumptions

Supplemental Table 1: Model assumptions

**Assumption**
Discount rate of 3%
Start point of simulation:<br>• Strategy "ERC": day of radical cystectomy<br>• Strategies involving NT: day of initial treatment delivery
Maximal age of 100 years
Patients do not decline RC after failed NT
Trimodal therapy is not an option after failed NT
Recurrences after RC are not detected earlier than 3 months postoperatively
ERC: early radical cystectomy; NT: novel therapy; RC: radical cystectomy

Simulation logic

The simulation logic describes the technical steps that are processed at different stages/events of the simulation. Parameters are described in the section "Input parameters". Due to technical reasons, some parameters are sampled in each patient although they might never be used. As an example, our model samples a time to muscle invasion after failure of novel therapy for each patient although not all simulated patients eventually experience failure of the novel therapy.

At start of simulation:

- *Strategy-independent tasks (before cloning)*
  - Sample age at start
  - Sample sex
  - Sample tumor type
  - Sample common random numbers for variance reduction
  - Sample time to all-cause mortality based on start age/sex and assign it to t_Death
- *Tasks specific to strategy "early radical cystectomy" (clone 1)*
  - Sample if patient has pT3/pT4 disease based on T3T4
  - Sample if patient has pN+ disease based on Nplus
  - If patient has pT3/pT4 and/or pN+ disease: sample if patients receives adjuvant chemotherapy based on p.AC (if yes: sample t_POC.Start based on t_RC.POC)
  - Sample if patient dies during radical cystectomy or postoperative recovery phase based on p.Death.RC (if yes: re-sample t_Death)
  - Sample time to recurrence after radical cystectomy (t_Recurrence)
    - Do not allow recurrence earlier than 3 months after surgery
    - Do not allow recurrence after C.Threshold
  - Set time to end of postoperative recovery phase (t_RC.End)
  - Set initial health state utility value to u.RC
- *Tasks specific to strategy "novel therapy (systemic)" (clone 2)*
  - Sample time to next cystoscopy/cytology visit (t_NT.Visit)
  - Sample time to failure of novel therapy (t_NT.Failure)
    - Do not allow failure after NT.Failure.Threshold
  - Sample time to muscle invasion after failure of novel therapy (t_NT.MIBC)
  - Sample time to metastatic disease after muscle invasion after failure of novel therapy (t_NT.Metastatic)
  - Set initial health state utility value to u.NT.active (systemic)
- *Tasks specific to strategy "novel therapy (low-intensity intravesical)" (clone 3)*
  - Set initial health state utility value to u.NT.active (low-intensity intravesical)
  - Otherwise identical to strategy "novel therapy (systemic)"
- *Tasks specific to strategy "novel therapy (high-intensity intravesical)" (clone 4)*
  - Set initial health state utility value to u.NT.active (high-intensity intravesical)
  - Otherwise identical to strategy "novel therapy (systemic)"

At event "NT.Visit":

- Cystocopy/cytology visit
- Check if event "NT.Failure" has occurred since the last visit:
  - Yes:

    - Sample time to transurethral resection of bladder tumor/bladder biopsy (t.NT.TURBT) based on d.TURBT
  - No:
    - Sample time to next cystoscopy/cytology visit (t_NT.Visit) based on d.NT.Visit
- Check if active treatment delivery of novel therapy is already over (based on d.NT.Duration):
  - Yes:
    - Set current health state utility value to u.NT.followup
  - No:
    - Keep current health state utility value (strategy-dependent)

At event "NT.Failure":

- Failure of novel therapy (biological event)
- No associated tasks

At event "NT.MIBC":

- Biological event that can occur in patients who failed novel therapy
- No associated tasks

At event "NT.TURBT":

- Transurethral resection of bladder tumor/bladder biopsy after detection of failed novel therapy during cystoscopy/cytology visit
- Check if event "NT.MIBC" has occurred before:
  - Yes:
    - Sample if patient receives neoadjuvant chemotherapy based on p.NAC:
      - Yes:
        - Sample time to start of neoadjuvant chemotherapy (t_POC.Start) based on d.TURBT.POC
        - Sample time to preoperative staging (t_NT.Staging) between current time point and t_POC.Start
      - No:
        - Sample time to radical cystectomy (t_RC.Start) based on d.TURBT.RC
        - Sample time to preoperative staging (t_NT.Staging) between current time point and t.RC.Start
  - No:
    - Sample time to radical cystectomy (t_RC.Start) based on d.TURBT.RC
    - Sample time to preoperative staging (t_NT.Staging) between current time point and t.RC.Start
- Modify health state utility value (if necessary)

At event "NT.Metastatic":

- Biological event that can occur in patients who failed novel therapy and progressed to muscle-invasive disease
- No associated tasks

At event “NT.Staging”:

- Preoperative staging before radical cystectomy in patients who failed novel therapy
- Check if event “NT.Metastatic” has occurred before:
    - Yes:
        - Change to a palliative management intention
        - Cancel radical cystectomy
        - Cancel any planned neoadjuvant chemotherapy
        - Modify t_Death according to t_Death.Recurrence.Local
    - No:
        - Continue with curative management intention
        - Leave event times unchanged
- Modify health state utility value (if necessary)

At event “POC.Start”:

- Start of perioperative chemotherapy
- Set any potential t_NT.MIBC or t_NT.Metastatic to missing
- Sample time to the end of perioperative chemotherapy (t_POC.End) based on d.POC
- If patient receives adjuvant chemotherapy: do not allow recurrence before chemotherapy is finished
- Sample if patient dies during perioperative chemotherapy based on p.Death.POC (if yes: re-sample t_Death)
- Modify health state utility value (if necessary)

At event “POC.End”:

- End of perioperative chemotherapy
- If patient received neoadjuvant chemotherapy: sample time to radical cystectomy (t_RC.Start) based on d.POC.RC
- Modify health state utility value (if necessary)

At event “RC.Start”:

- Radical cystectomy/start of postoperative recovery phase
- Set any potential t_NT.MIBC or t_NT.Metastatic to missing
- Sample if patient dies during radical cystectomy or postoperative recovery phase based on p.Death.RC (if yes: re-sample t_Death)
- Sample if patient has pT3/pT4 disease based on T3T4
- Sample if patient has pN+ disease based on Nplus
- If patient has pT3/pT4 and/or pN+ disease and did not receive neoadjuvant chemotherapy: sample if patients receives adjuvant chemotherapy based on p.AC (if yes: sample t_POC.Start based on t_RC.POC)
- Set time to end of postoperative recovery phase (t_RC.End)
- Check if event “NT.Metastatic” has occurred before:
    - Yes:
        - Sample time to recurrence after radical cystectomy (t_Recurrence) as 3 months +/- 7 days
    - No:
        - Sample time to recurrence after radical cystectomy (t_Recurrence)

    - Do not allow recurrence earlier than 3 months after surgery
    - Do not allow recurrence after C.Threshold
- Set initial health state utility value to u.RC

At event "RC.End":

- End of postoperative recovery phase
- Modify health state utility value (if necessary)

At event "Recurrence":

- Recurrence after radical cystectomy
- Sample if patient experiences a local recurrence (with or without systemic recurrence) after radical cystectomy based on p.local.recurrence:
    - Yes: Modify t_Death according to t_Death.Recurrence.Local
    - No: Modify t_Death according to t_Death.Recurrence.NoLocal
- Modify health state utility value (if necessary)

At event "Death":

- Remove patient from simulation

Input parameters

Patient characteristics at start of simulation

- Age at start:
  - Truncated normal distribution:
    - Mean: 70.5 years
    - Standard deviation: 9.63 years
    - Lower truncation: 40 years
    - Upper truncation: 90 years)
  - Based on: Shore et al. [1]
- Sex:
  - Female: 17.5%
  - Male: 82.5%
  - Based on: Shore et al. [1]
- Tumor type:
  - Empirical frequency distribution:
    - Carcinoma in situ only: 52.5%
    - Ta only: 10%
    - Ta with concurrent carcinoma in situ: 10%
    - T1 only: 15%
    - T1 with concurrent carcinoma in situ: 12.5%
  - Based on: Shore et al. [1]

Time to event parameters

Supplemental Table 2: Time to event parameters

Event	Parameter/distribution	Source
Time to all-cause mortality in general population	<u>Weibull distribution</u><br><br>Weibull parameters were estimated for each age at start and sex based on empirical distributions derived from life tables.<br><br>Calibrated	Life tables provided by the National Center for Health Statistic of the United States [2]
Time to next cystoscopy/cytology visit (t_NT.Visit)	3 months in the first 2 years<br>6 months in years 3 and 4<br>12 months after year 4<br><br>Exact timing is allowed to vary between -7 days to +7 days.	Common practice in high-risk non-muscle invasive bladder cancer (Babjuk et al. [3])

**Event**	**Parameter/distribution**	**Source**
Duration of active treatment delivery (d.NT.Duration)	Systemic:<br>• 8 visits*<br>• 2 years<br><br>Low-intensity intravesical:<br>• 4 visits*<br>• 1 year<br><br>High-intensity intravesical:<br>• 1 visit*<br>• 3 months<br><br>*cystocscopy/cystology visits	Assumed durations (see *Table 1* of main article)
Time to NT failure (t_NT.Failure)	<u>Piece-wise exponential distributions with 6 pieces</u><br><br>Parameters were derived by using an optimization algorithm (simulated annealing [4]) to find piece-wise exponential distributions that match the recommendations of the IBCG and FDA/AUA (including their transformations +5%, +10%, and +15%) as close as possible. More details can be found in the main article and in Section “Failure curves” of the *Supplemental Appendix*.	IBCG (Kamat et al. [5]) and FDA/AUA (Jarow et al. [6]) recommendations
Threshold after which t_NT.Failure is not allowed anymore (NT.Failure.Threshold)	7.5 years	Matusomoto et al. [7] and Fernandez-Gomez et al. [8]
Interval between detection of NT failure and TURBT (d.TURBT)	<u>Uniform distribution</u><br><br>1 week to 6 weeks	Clinical practice/experience
Interval between TURBT and RC (d.TURBT.RC)	<u>Uniform distribution</u><br><br>4 weeks to 6 weeks	Clinical practice/experience
Interval between TURBT and neoadjuvant chemotherapy (d.TURBT.POC)	<u>Uniform distribution</u><br><br>1 week to 2 weeks	Clinical practice/experience
Interval between end of neoadjuvant chemotherapy and RC (d.POC.RC)	<u>Uniform distribution</u><br><br>3 weeks to 6 weeks	Clinical practice/experience

**Event**	**Parameter/distribution**	**Source**
Time to muscle invasion after failure of novel therapy (t_NT.MIBC)	Exponential distribution<br><br>rate: 0.69; unit: years<br><br>Based on a median time of 1 year.<br><br>The uncertainty associated with this parameter was addressed probabilistically in the final analysis. We allowed for uniformly distributed median times between 0.25 years and 1.7 years.	Expert opinion bounded by extrapolations from related but not readily comparable literature
Time to metastatic disease after muscle invasion after failure of novel therapy (t_NT.Metastatic)	2-step sampling:<br>1. Sample time from muscle invasion to death if patient does not receive any treatment (Weibull distribution derived by using an optimization algorithm (simulated annealing [4]) to find the Weibull distribution that matches a median time of 1 year and 1% survival at 2 years as close as possible).<br>2. Subtract a value sampled from a uniform distribution ranging from 1 day to the value sampled in step 1.<br><br>The uncertainty associated with these parameters was addressed probabilistically in the final analysis. We allowed for uniformly distributed median times between 0.5 years and 1.5 years as well as for 1% survival times between 1 year and 3 years.	Expert opinion bounded by extrapolations from related but not readily comparable literature
Duration of postoperative recovery phase (t_RC:End)	3 months	Clinical practice/common definition
Interval between RC and adjuvant chemotherapy (t_RC.POC)	Uniform distribution<br><br>6 weeks to 12 weeks	Clinical practice

Event	Parameter/distribution	Source
Duration of perioperative chemotherapy (d.POC)	DDMVAC3: 42 days<br>DDMVAC4: 56 days<br>GEMCIS21: 84 days<br>GEMCIS28: 112 days<br><br>If patient is sampled to receive perioperative chemotherapy, he/she receives one of the 4 regimens (25% probability to be sampled for each).	Clinical practice
Time to recurrence after RC (t_Recurrence)	Weibull distribution<br><br>lambda: 11.23; gamma 0.66; unit: years<br><br>Modified by hazard ratio derived from a Cox proportional hazards regression model accounting for:<br>• Sex<br>• Age at RC<br>• pT stage at RC<br>• pN stage at RC<br>• CIS at RC<br>• Receipt of neoadjuvant chemotherapy<br>• Receipt of adjuvant chemotherapy<br><br>Weibull parameters were estimated by fitting a parametric survival model to reconstructed patient-level data. We therefore used the reconstruction algorithm developed by Guyot et al. [16]. This algorithm requires both the coordinates of the Kaplan-Meier estimator as well as the associated number at risk table. The extraction of the coordinates was performed by an established semi-automated webtool (WebPlotDigitizer).<br><br>Calibrated (lambda, gamma, and hazard ratio)	Karakiewicz et al. [9]<br>Individual-level data were reconstructed from Figure 1A.
Threshold after which t_Recurrence is not allowed anymore (C.Threshold)	5 years<br><br>Calibrated	Herr et al. [10]
Time to death after patient experiences local recurrence after RC (with or without systemic recurrence) (t_Death.Recurrence.Local)	Exponential distribution<br><br>rate: 1.39; unit: years<br><br>Based on a median time of 6 months.	Reported median time between 4 months and 8 months (Witjes et al. [11])

Event	Parameter/distribution	Source
Time to death after patient experiences non-local recurrence after RC (systemic recurrence only) (t_Death.Recurrence.NoLocal)	Exponential distribution<br><br>rate: 0.48; unit: years<br><br>Based on a median time of 17.5 months.	Reported median time between 9 months and 26 months (Witjes et al. [11])

AUA: American Urology Association; CIS: carcinoma in situ; DDMVAC3/4: 3 or 4 cycles of dose-dense methotrexate-vinblastine-adriamycin-cisplatin; FDA: United States Food and Drug Administration; GEMCIS21/28: 21 days or 28 days of gemcitabine-cisplatin; IBCG: International Bladder Cancer Group; MIBC: muscle-invasive bladder cancer; NT: novel therapy; POC: perioperative chemotherapy; RC: radical cystectomy; TURBT: transurethral resection of bladder tumor

Probabilities

Supplemental Table 3: Probabilities

Event	Probability	Source
Neoadjuvant chemotherapy if patient is diagnosed with muscle-invasive bladder cancer (p.NAC)	27%	Booth et al. [12]
Mortality during RC (p.death.RC)	Logistic regression model accounting for:<br>• Age at RC<br>• Nodal status at RC<br>• Metastatic disease at RC)	Aziz et al. [13]
Presence of pT3/pT4 disease at RC (T3T4)	Logistic regression model accounting for:<br>• Age at RC<br>• Female sex<br>• Presence muscle-invasive disease at prior TURBT<br>• Presence of carcinoma in situ at prior TURBT<br>• Receipt of neoadjuvant chemotherapy)<br><br>Calibrated	Karakiewicz et al. [14]
Presence of pN+ disease at RC (Nplus)	Logistic regression model accounting for:<br>• Age at RC<br>• Female sex<br>• Presence muscle-invasive disease at prior TURBT<br>• Presence of carcinoma in situ at prior TURBT<br>• Receipt of neoadjuvant chemotherapy<br><br>Calibrated	Karakiewicz et al. [14]
Adjuvant chemotherapy if patient is diagnosed with pT3/pT4 and/or pN+ at RC (p.AC)	30%	Booth et al. [12]
Mortality during neoadjuvant or adjuvant chemotherapy (p.Death.POC)	1%	Expert opinion
Local recurrence if patient experiences recurrence (p.local.recurrence)	Logistic regression model accounting for:<br>• Receipt of neoadjuvant chemotherapy<br>• Presence of pT3/pT4 disease at RC<br>• Presence of pN+ disease at RC<br>• Presence of positive surgical margins at RC	Herr et al. [15]
AC: adjuvant chemotherapy; NAC: neoadjuvant chemotherapy; POC: perioperative chemotherapy; RC: radical cystectomy; TURBT: transurethral resection of bladder tumor		

Utilities

Supplemental Table 4: Health state utility values

Health state	Utility	Source
Regular follow-up after active treatment delivery of NT [u.NT.followup]	1	Reference value
Active treatment delivery of NT (systemic) [u.NT.active]	0.90	Pooled value of Aguiar et al. [16] and Kohn et al. [17] based on pembrolizumab usage in a responsive setting
Active treatment delivery of NT (low-intensity intravesical) [u.NT.active]	0.98	Kulkarni et al. [18] based on BCG therapy
Active treatment delivery of NT (high-intensity intravesical) [u.NT.active]	0.90	DiSantostefano et al. [19] based on mild BPH urinary symptoms
Post-cystectomy state [u.PC]	0.94	Kulkarni et al. [18]
RC and postoperative recovery phase [u.RC]	0.80	Kulkarni et al. [18]
Perioperative chemotherapy [u.POC]	0.64	Kulkarni et al. [18]
Non-local recurrence (systemic recurrence only) after RC [u.non.local.recurrence]	0.62	Kulkarni et al. [18]
Local recurrence (with or without systemic recurrence) after RC [u.local.recurrence]	0.30	Kulkarni et al. [18]

BCG: Bacillus Calmette-Guérin; BPH: benign prostatic hyperplasia; NT: novel therapy; PC: post-cystectomy; POC: perioperative chemotherapy; RC: radical cystectomy

We addressed the uncertainty associated with the health state utility values probabilistically in the final analysis. To maintain clinical validity (e.g. u.NT.active > u.local.recurrence), we used an ordered sampling approach as proposed by *Ren et al.* [20]. The variance required to sample each utility value was defined as $[(\text{utility} * 1.1 - \text{utility}) / 2]^2$.

Validation and calibration

External validation methodology

As part of the validation process, we compared the outputs of our model of the strategy "early radical cystectomy" to outcomes reported in literature (validation targets). To fulfil the criteria of a formal external validation, the studies used to inform the validation targets were not allowed to be part of the input sources. Furthermore, simulated patients, in contrast to regular study participants, are never lost to follow-up. To account for this important difference, the external validation approach had to mimic the censoring patterns observed in the studies that we used to inform the validation targets. This was implemented by sampling censoring times from gamma distributions as described by *Wallis et al.* [21]. The validation targets are listed in *Supplemental Table 5.*

Supplemental Table 5: Validation targets

Validation target	Reported value	Source
pT3/pT4 disease at early radical cystectomy	13%	Haas et al. [22]
Positive nodal disease at early radical cystectomy	13%	Haas et al. [22]
Cancer-specific survival at 5 years	85%	Haas et al. [22]
Cancer-specific survival at 10 years	76%	Haas et al. [22]

Calibration methodology

We calibrated several input parameters as we detected a certain deviation of the initial model output from the validation targets. The calibration process was divided into two steps. In a first step, we calibrated our model to meet the proportions of pT3/pT4 disease and positive nodal disease reported in literature while the second step focused on calibrating the model against cancer-specific survival at 5 and 10 years.

Step 1
The proportions of pT3/pT4 disease and positive nodal disease are determined by the corresponding multivariable logistic regression models. Therefore, we modified the output of the two regression models with calibration factors which means that, at each calculation, the preliminary probabilities are multiplied by specific calibration factors (numerical values between 0 and plus infinity). A calibration factor of 1 means no calibration while values below 1 and above 1 translate into a decrease and an increase of the preliminary probability, respectively. An optimal set of calibration factors was defined as the parameter set that minimizes the difference between model output and validation targets. We quantified this difference by a single numeric value, the weighted goodness of fit measure, that simultaneously evaluates the deviation of several model output/validation target pairs (see *Vanni et al.* [23]). The lower the weighted goodness of fit measure, the closer the set of calibration factors matches the validation targets.

To find an optimal set of calibration factors, we used the following optimization approach:

1. Define a plausibility range for each calibration factor
2. Sample several sets of calibration factors (usually hundreds/thousands). To enhance the coverage of the whole parameter space, we used latin hypercube sampling [23].

3. Run the simulation with each set of calibration factors and record the resulting weighted goodness of fit measure
4. Restrict the sets of calibration factors to the ones that yield weighted goodness of fit measures below the first percentile (see *Supplemental Figure 4*)
5. Validity of model output:
    a. Satisfying: Integrate each of the sets obtained in step 4 in a probabilistic way into the final analysis by weighted sampling (weights = 1 / goodness of fit measure)
    b. Unsatisfying: Go back to step 1 and refine the plausibility ranges according to the ranges of the sets obtained in step 4

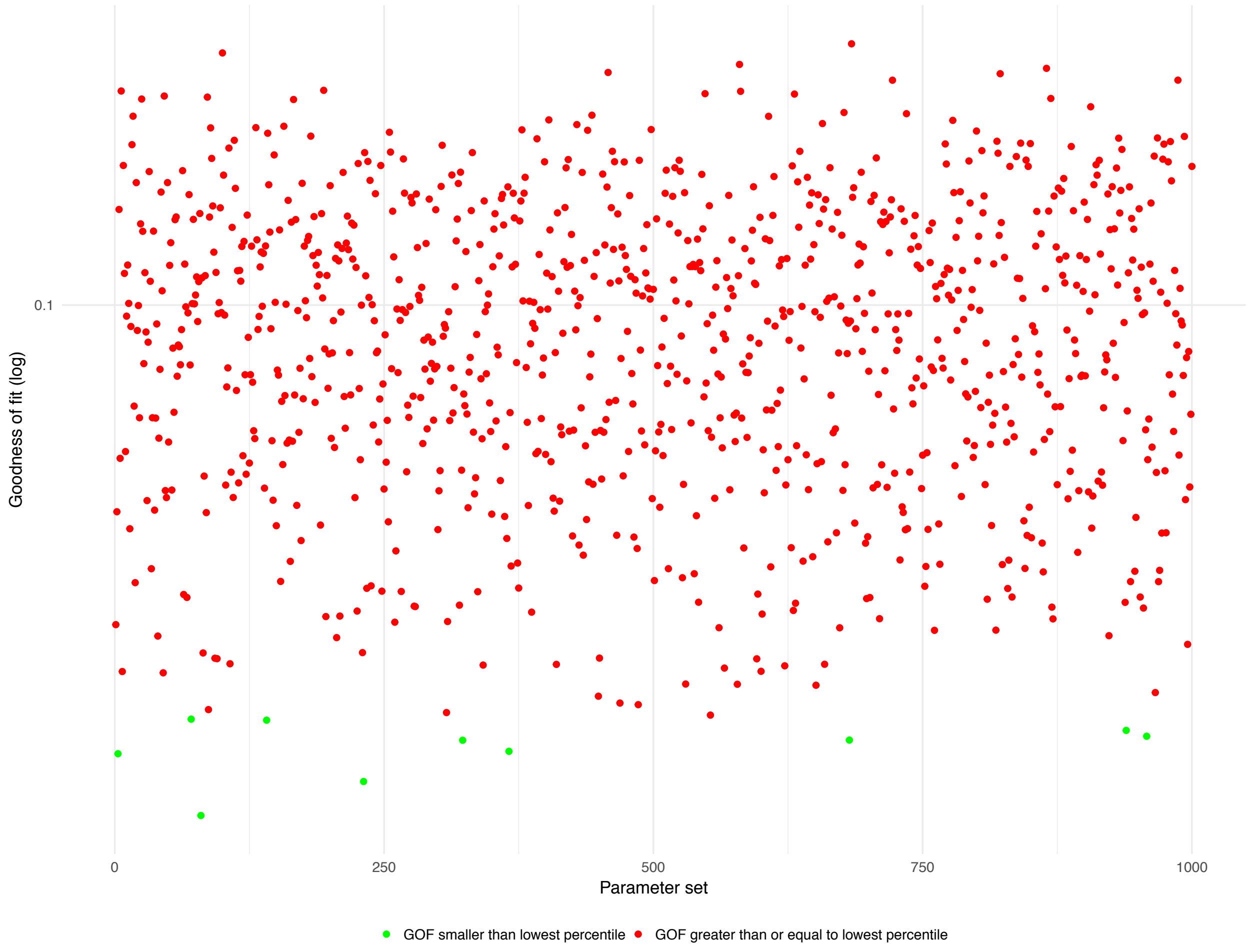

Supplemental Figure 4: Red dots represent sets of calibration factors that yielded weighted goodness of fit measures below the first percentile.

After several iterations of the above-mentioned optimization algorithm, we identified a set of calibration factors that yielded a model output with only minimal deviation from the validation targets. We therefore considered our model valid with regards to proportions of pT3/pT4 disease and positive nodal disease.

Step 2

Next, we calibrated our model against cancer-specific survival at 5 and 10 years as reported in literature. We assumed these validation targets to be highly influenced by the background mortality and time to recurrence after radical cystectomy (t_Recurrence). The former was calibrated by a simple calibration factor as described earlier while the calibration of the latter was decomposed into:

- Calibration of lambda parameter of Weibull distribution
- Calibration of gamma parameter of Weibull distribution
- Calibration of hazard ratio modifying the raw event time yielded by the Weibull distribution
- Calibration of C.Threshold (threshold after which t_Recurrence is not allowed anymore)

To find an optimal set of input parameters, we used the optimization approach as described earlier. After both calibration steps, our model output matched the results reported in literature very closely (see *Supplemental Table 6*). We therefore considered it valid.

Supplemental Table 6: Deviation of model output from results reported in literature (after calibration)

Validation target	Model output	Literature
pT3/pT4 disease at early radical cystectomy	15.4%	13%
Positive nodal disease at early radical cystectomy	13.2%	13%
Cancer-specific survival at 5 years	86.6%	85%
Cancer-specific survival at 10 years	76.4%	76%

Failure curves

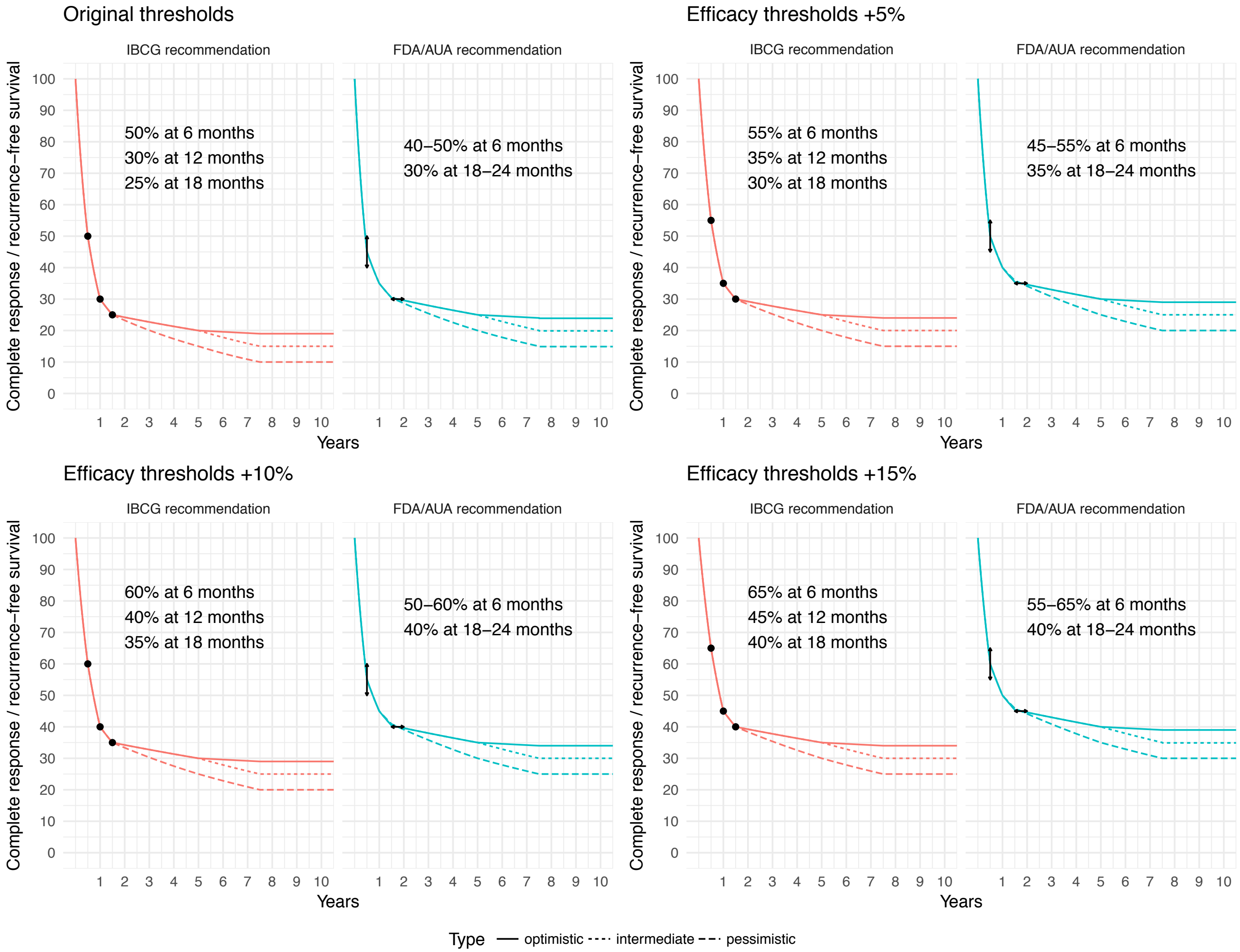

Supplemental Figure 5: Failure curves reflecting different efficacy thresholds. *AUA: American Urology Association; FDA: United States Food and Drug Administration; IBCG: International Bladder Cancer Group*

Supercomputer configuration

We used the following simulation architecture:

- Lower level: simulation of each strategy among a cohort of 100,000 patients (4 x 100,000 patients)
- Middle level: replication (1,000 times) of each lower-level run to reflect the uncertainty associated with some input parameters (expert opinions, health state utility values, and parameters derived through calibration)
- Upper level: simulation of the 24 efficacy thresholds

The cohort size of 100,000 patients was chosen empirically as this number yielded highly stable results with a percentage deviation of less than 1% from the mean value (see *Supplemental Figure 6*). The reliable analysis of 24 efficacy thresholds required simulating the clinical course of 9.6 billion individuals (4 strategies x 100,000 patients x 1,000 probabilistic samples x 24 efficacy thresholds). From a computational perspective, the *simmer* simulation core [24] had to be fed with 24,000 input sets (1,000 probabilistic samples x 24 efficacy thresholds).

The computations were performed on the Niagara supercomputer at the SciNet HPC Consortium [25, 26]. All simulation runs were performed during a resource allocation window that provided 640 computation cores (on 16 nodes) for 24 hours (effective computation time: 19 hours and 12 minutes). Each node consisted of 40 Intel Skylake cores at 2.4 GHz and 202 GB RAM. We distributed each lower-level simulation run (4 x 100,000 patients) across 10 sub-runs (4 x 10,000 patients) to prevent a memory overload although this increased the number of times the *simmer* simulation core [24] had to be initialized to 240,000 (10 sub-runs x 1,000 probabilistic samples x 24 efficacy thresholds). The resulting 240,000 inputs sets were delivered in chunks of 160 to the 16 nodes. Within each node, the 160 input sets of a single chunk were distributed across 40 cores so they could be processed in parallel.

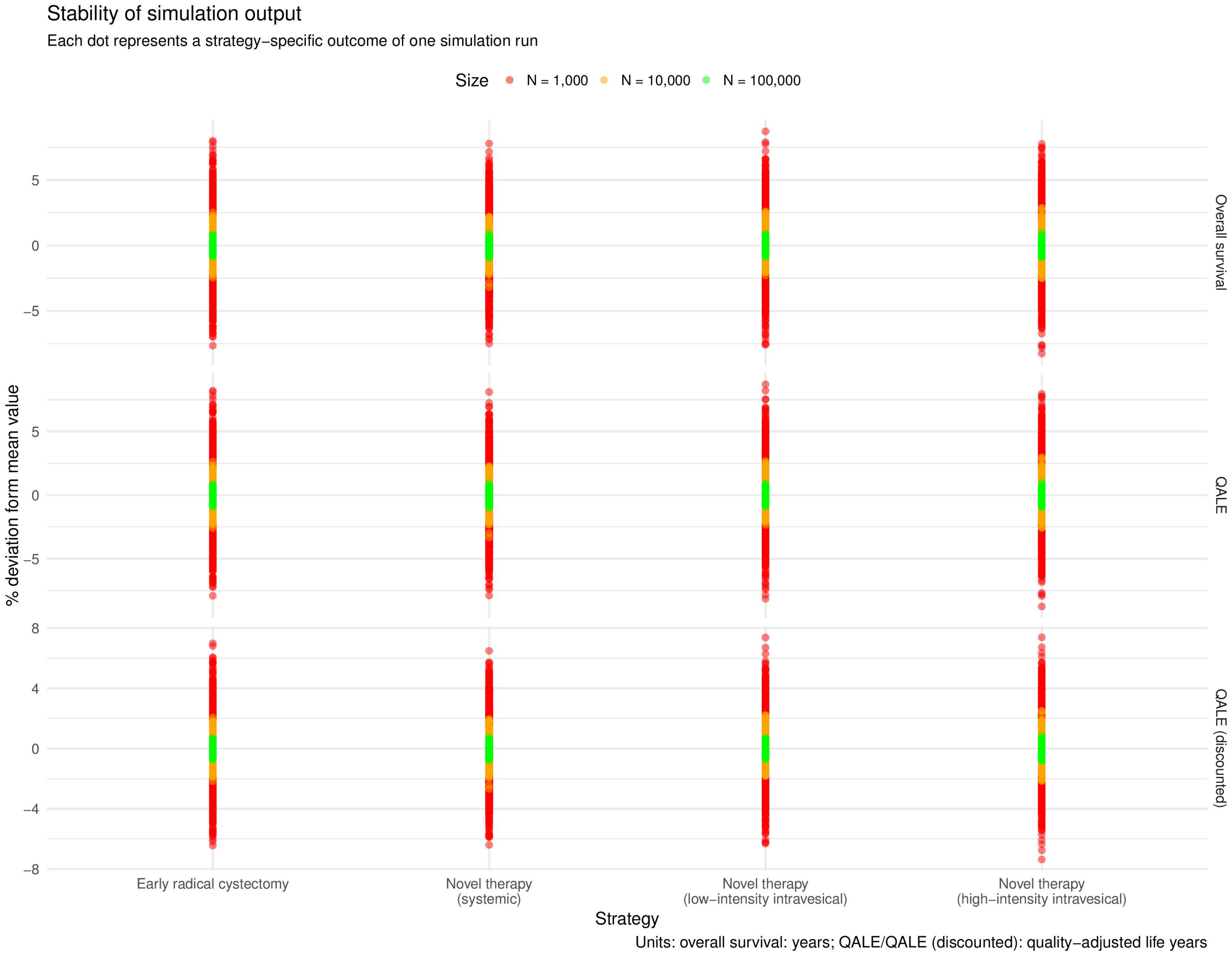

Supplemental Figure 6: Stability of simulation output. *QALE: Quality-adjusted life expectancy*

References

[1] Shore ND, Boorjian SA, Canter DJ, et al. Intravesical rAd-IFNalpha/Syn3 for Patients With High-Grade, Bacillus Calmette-Guerin-Refractory or Relapsed Non-Muscle-Invasive Bladder Cancer: A Phase II Randomized Study. J Clin Oncol. 2017;35:3410-6.
[2] Arias E, Xu J. United States Life Tables, 2015. Natl Vital Stat Rep. 2018;67:1-64.
[3] Babjuk M, Bohle A, Burger M, et al. EAU Guidelines on Non-Muscle-invasive Urothelial Carcinoma of the Bladder: Update 2016. Eur Urol. 2017;71:447-61.
[4] Xiang Y, Gubian S, Suomela B, Hoeng J. Generalized Simulated Annealing for Global Optimization: The GenSA Package. R Journal. 2013;5.
[5] Kamat AM, Sylvester RJ, Bohle A, et al. Definitions, End Points, and Clinical Trial Designs for Non-Muscle-Invasive Bladder Cancer: Recommendations From the International Bladder Cancer Group. J Clin Oncol. 2016;34:1935-44.
[6] Jarow JP, Lerner SP, Kluetz PG, et al. Clinical trial design for the development of new therapies for nonmuscle-invasive bladder cancer: report of a Food and Drug Administration and American Urological Association public workshop. Urology. 2014;83:262-4.
[7] Matsumoto K, Kikuchi E, Horiguchi Y, et al. Late recurrence and progression in non-muscle-invasive bladder cancers after 5-year tumor-free periods. Urology. 2010;75:1385-90.
[8] Fernandez-Gomez J, Madero R, Solsona E, et al. Predicting nonmuscle invasive bladder cancer recurrence and progression in patients treated with bacillus Calmette-Guerin: the CUETO scoring model. J Urol. 2009;182:2195-203.
[9] Karakiewicz PI, Shariat SF, Palapattu GS, et al. Nomogram for predicting disease recurrence after radical cystectomy for transitional cell carcinoma of the bladder. J Urol. 2006;176:1354-61; discussion 61-2.
[10] Herr HW, Sogani PC. Does early cystectomy improve the survival of patients with high risk superficial bladder tumors? J Urol. 2001;166:1296-9.
[11] Alfred Witjes J, Lebret T, Comperat EM, et al. Updated 2016 EAU Guidelines on Muscle-invasive and Metastatic Bladder Cancer. Eur Urol. 2017;71:462-75.
[12] Booth CM, Karim S, Brennan K, Siemens DR, Peng Y, Mackillop WJ. Perioperative chemotherapy for bladder cancer in the general population: Are practice patterns finally changing? Urol Oncol. 2018;36:89 e13-89 e20.
[13] Aziz A, May M, Burger M, et al. Prediction of 90-day mortality after radical cystectomy for bladder cancer in a prospective European multicenter cohort. Eur Urol. 2014;66:156-63.
[14] Karakiewicz PI, Shariat SF, Palapattu GS, et al. Precystectomy nomogram for prediction of advanced bladder cancer stage. Eur Urol. 2006;50:1254-60; discussion 61-2.
[15] Herr HW, Faulkner JR, Grossman HB, et al. Surgical factors influence bladder cancer outcomes: a cooperative group report. J Clin Oncol. 2004;22:2781-9.
[16] Aguiar PN, Jr., Perry LA, Penny-Dimri J, et al. The effect of PD-L1 testing on the cost-effectiveness and economic impact of immune checkpoint inhibitors for the second-line treatment of NSCLC. Ann Oncol. 2017;28:2256-63.
[17] Kohn CG, Zeichner SB, Chen Q, Montero AJ, Goldstein DA, Flowers CR. Cost-Effectiveness of Immune Checkpoint Inhibition in BRAF Wild-Type Advanced Melanoma. J Clin Oncol. 2017;35:1194-202.
[18] Kulkarni GS, Finelli A, Fleshner NE, Jewett MA, Lopushinsky SR, Alibhai SM. Optimal management of high-risk T1G3 bladder cancer: a decision analysis. PLoS Med. 2007;4:e284.

[19] DiSantostefano RL, Biddle AK, Lavelle JP. The long-term cost effectiveness of treatments for benign prostatic hyperplasia. Pharmacoeconomics. 2006;24:171-91.
[20] Ren S, Minton J, Whyte S, Latimer NR, Stevenson M. A New Approach for Sampling Ordered Parameters in Probabilistic Sensitivity Analysis. Pharmacoeconomics. 2018;36:341-7.
[21] Wallis CJD, Morton G, Jerath A, et al. Adjuvant Versus Salvage Radiotherapy for Patients With Adverse Pathological Findings Following Radical Prostatectomy: A Decision Analysis. MDM Policy & Practice. 2017;2:2381468317709476.
[22] Haas CR, Barlow LJ, Badalato GM, DeCastro GJ, Benson MC, McKiernan JM. The Timing of Radical Cystectomy for bacillus Calmette-Guerin Failure: Comparison of Outcomes and Risk Factors for Prognosis. J Urol. 2016;195:1704-9.
[23] Vanni T, Karnon J, Madan J, et al. Calibrating models in economic evaluation: a seven-step approach. Pharmacoeconomics. 2011;29:35-49.
[24] Ucar I, Smeets B, Azcorra A. simmer: Discrete-Event Simulation for R. Journal of Statistical Software. 2019;90:30.
[25] Loken C, Gruner D, Groer L, et al. SciNet: Lessons Learned from Building a Power-efficient Top-20 System and Data Centre. Journal of Physics: Conference Series. 2010;256: 012026.
[26] Ponce M, Zon Rv, Northrup S, et al. Deploying a Top-100 Supercomputer for Large Parallel Workloads: the Niagara Supercomputer. Proceedings of the Practice and Experience in Advanced Research Computing on Rise of the Machines (learning). Chicago, IL, USA: ACM, 2019:1-8.
